# Research note: Occurrence of *mcr-*encoded colistin resistance in *Escherichia coli* from pigs and pig farm workers in Vietnam

**DOI:** 10.1093/femsmc/xtaa003

**Published:** 2020-10-09

**Authors:** Son Thi Thanh Dang, Duong Thi Quy Truong, John Elmerdahl Olsen, Nhat Thi Tran, Giang Thi Huong Truong, Hue Thi Kim Vu, Anders Dalsgaard

**Affiliations:** National Institute of Veterinary Research, 74 Truong Chinh, Phuong Dinh, Dong Da, Hanoi, Vietnam; National Institute of Veterinary Research, 74 Truong Chinh, Phuong Dinh, Dong Da, Hanoi, Vietnam; Department of Veterinary and Animal Sciences, Faculty of Health and Medical Sciences, University of Copenhagen, Stigboejlen 4, Frederiksberg C 1870, Denmark; National Institute of Veterinary Research, 74 Truong Chinh, Phuong Dinh, Dong Da, Hanoi, Vietnam; National Institute of Veterinary Research, 74 Truong Chinh, Phuong Dinh, Dong Da, Hanoi, Vietnam; National Institute of Veterinary Research, 74 Truong Chinh, Phuong Dinh, Dong Da, Hanoi, Vietnam; Department of Veterinary and Animal Sciences, Faculty of Health and Medical Sciences, University of Copenhagen, Stigboejlen 4, Frederiksberg C 1870, Denmark; School of Chemical and Biomedical Engineering Nanyang Technological University, 62 Nanyang Drive Singapore 637459

**Keywords:** colistin resistance, resistance gens, *E. coli*, pig feces, farm-worker, Vietnam

## Abstract

WHO considers colistin as a highest priority critically important drug for human health, and occurrence of colistin-resistant bacteria in livestock is of health concern. The current study determined occurrence of colistin-resistant *Escherichia coli* in pigs and workers at pig farms in Vietnam, and investigated the genetic background for resistance. Colistin-resistant *E. coli* were detected from pigs in 53/116 (45.7%) farms, and from workers taking care of the pigs in 21/94 (22.3%) farms. Colistin-resistant isolates showed MIC to colistin between 4–16 mg/L, they were multidrug resistant (99%) and resistance was caused by the presence of *mcr-1* genes in 97/102 (95.1%) *E. coli* from pigs and in 31/34 (91.1%) isolates from humans. *mcr-1* is considered a plasmid-encoded gene, but this was not confirmed in the current investigation. In total, one pig isolate carried both *mcr-1* and *mcr-3* genes, whereas *mcr-2, mcr-4* and *mcr-5* genes were not detected. Shared resistance profiles between pig and human isolates on the same farm was only observed in four farms. The study showed that commensal *E. coli* from pigs in Vietnam constitute a reservoir for colistin-resitant *E. coli*, however, further studies are needed to confirm that *mcr* genes are associated with plasmids and their importance for human health.

## Teaser

Commensal *E. coli* from pigs and pig farm workers in Vietnam constitute a main reservoir for colistin-resistant *E. coli*.

## INTRODUCTION

Antimicrobial resistance (AMR) is a global health threat (World Health Organization 2015), and both human use and livestock farming contribute to the AMR burden (Chereau *et al*. 2017). Antimicrobials are commonly used in pig production in Vietnam for disease prevention, treatment of diseases and until recently also for growth promotion, leading to an increased risk of AMR (Dang *et al*. 2011; Lai *et al*. 2014). The World Health Organization (WHO) has raised a special concern over the use of highest-priority critically important antimicrobials (HP-CIAs) in livestock (World Health Organization 2017a), since several drugs belonging to this category are widely used for animal production, especially for pig production (Bloom *et al*. 2017).

*Escherichia coli* is a commensal bacteria of the gastrointestinal tract of animals and humans. It is widely used as an indicator organism to monitor AMR in livestock, food and people (Rasheed *et al*. 2014; Singh *et al*. 2018; Pormohammad, Nasiri and Azimi 2019). Most studies of AMR in livestock have been conducted in intensive-production systems in developed countries (Cho *et al*. 2011; Delannoy *et al*. 2017; Randall *et al*. 2018; Singh *et al*. 2018; Zhang *et al*. 2018; Yamamoto *et al*. 2019), and less data are available from semi-intensive systems prevalent in low and middle income countries, where farmers and veterinarians have limited knowledge on prudent antimicrobial use (AMU) practices. In Vietnam, studies on the prevalence of AMR among *E. coli* strains from livestock (Nguyen et al. 2015, 2016; Nhung *et al*. 2015) have not had a particular focus on HP-CIAs.

Colistin is a HP-CIAs antimicrobial, as it is a last resort drug for treatment of systemic infections with multi-drug resistant Gram-negative bacteria in humans (World Health Organization 2017a). Despite this, it is still anticipated commonly used in pig production in Vietnam, even for preventive measures. The emergence of mobile colistin resistance genes (*mcr*) on plasmids is of concern because of rapid spread of colistin resistance within and between bacterial populations. Three different *mcr* genes (*mcr-1, mcr-2* and *mcr-3*) were originally described on plasmids in *Enterobacteriaceae* (Xavier *et al*. 2016; Hernández *et al*. 2017; Randall *et al*. 2018; Zhang *et al*. 2018), and later more genes, including the *mcr-4* and *mcr-5* genes have been added (Zhang *et al*. 2018; Ström Hallenberg *et al*. 2019).

The objective of this study was to determine the prevalence of colistin-resistant *E. coli* in pigs and workers at pig farms in Northern Vietnam and to determine whether *mcr* genes were present among *E. coli* from these sources.

## MATERIAL AND METHOD

### Fecal sampling

Composite pig manure samples were collected at 116 randomly selected pig farms from four districts in Bac Ninh province, including 31 small-scale farms (≤ 20 pigs/farm), 66 middle- scale farms (21–99 pigs/farm) and 19 large-scale farms (≥ 100 pigs/farm). The farms were selected based on lists provided by the provincial Department of Animal Health. Piglets raised in middle-scale and large-scale farms were mainly provided by one local large pig company, whereas small-scale farms kept sows and produced their own piglets. Consent was sought and obtained to collect faecal samples from workers at 94 of the pig farms. Briefly, 100–200 g faecal matter was collected with a spoon from the floor of different pens and sizes of pigs. A faecal sample was self-collected by one worker (approximately 20 g) during defecation using sterile gloves on the same day as the pigs were sampled. The samples were immediately placed in labeled sterile plastic bags, mixed by manual handling and transported to the laboratory, where they were analyzed the same day. The farm owner and farm workers gave consent orally and understood that they could withdraw from the study at any time and that they would be anonymous in the reporting of the results. The study protocol was approved by the Ethical Committee of the National Institute of Nutrition in Hanoi (Certificate No. 04/VDD-QLKH).

### Isolation of colistin-resistant *E. coli*

A total of 10 g of faecal sample were mixed with 90 mL Peptone Saline Buffer containing 0.1% peptone (Himedia, Mumbai, India) and 0.85% NaCl (Merck, Darmstadt, Germany) in a sterile plastic bag and homogenized in a stomacher. A 10-µL loop of the dilution was spread onto MacConkey agar (Merck, Darmstadt, Germany) containing 4 mg/L colistin (Sigma, St Louis, MO). The agar plate was incubated at 37°C for 24 h for the selection of presumptive colistin-resistant *E. coli*. Up to five presumptive *E. coli* colonies (red, smooth, round, >2.5 mm) were selected from each plate showing bacterial growth. Isolates were confirmed as *E. coli* by biochemical tests including glucose (+), lactose (+), gas (+), H2S (−), indole (+), urease (−), methyl red (+) and citrate (−) tests. *E. coli* ATCC25922 was used as control strain.

### Antimicrobial susceptibility testing

All confirmed *E. coli* growing on the MacConkey agar containing 4 mg/L colistin were tested for susceptibility to 12 antimicrobials including: ampicillin (AMP, 10 µg); gentamycin (GEN, 10 µg), trimethoprim (TMP, 5 µg), tetracycline (TET, 30 µg), streptomycin (STR, 10 µg), nalidixic acid (NAL, 30 µg), sulfonamides (SUL, 300 µg), ciprofloxacin (CIP, 5 µg), ceftriaxone (CRO, 30 µg), cefoxitin (FOX, 30 µg), ceftiofur (EFT, 30 µg) and amoxicillin-clavulanic acid (AMC, 30 µg), by Kirby–Bauer disk diffusion assay (Hudzicki 2009). *E. coli* suspected of producing extended spectrum β-lactamases (ESBL) were confirmed via the double-disk synergy test (Naseer^2017^). Micro-broth dilution assay was used to confirm colistin (COL) resistance, using 2-fold dilutions of colistin sulfate (Sigma Aldrich, St Louis, MO). *E. coli* ATCC 25 922 was used as a control strain. Interpretation of antimicrobial susceptibility test results was done according to CLSI criteria (Clinical and Laboratory Standards Institute (CLSI) 2017). Isolates classified as intermediate resistant were categorized as susceptible to avoid overestimation of resistance. Multi-drug resistant (MDR) was defined as resistance to at least one antimicrobial in three antimicrobial classes (Magiorakos *et al*. 2012).

### Detection of colistin resistance genes

Colistin resistance encoded by *mcr-1, mcr-2, mcr-3, mcr-4* and *mcr-5* genes was detected by multiplex PCR (Table 1; Rebelo *et al*. 2018). Each PCR reaction contained 12.5 µL DreamTaq Green PCR Master Mix 2X (Thermo-Fisher, Waltham, MA), five primer pairs (DNA-Diagnostic, Denmark), 5.5 µL nuclease-free water and 2 µL DNA template obtained by using a DNA isolation kit (Thermo-Fisher). The PCR reactions were carried out with the following amplification conditions: an initial denaturation step at 94°C for 15 min, followed by 35 cycles of denaturation (94°C for 30 s), annealing (58°C for 90 s) and extension (72°C at 60 s), followed by a final extension at 72°C for 10 min on a PCR thermal cycler (Biorad, Hercules, CA). Positive strains were included for each gene (Table 1). The same PCR reactions without template DNA was included as negative controls in all PCR reactions. The PCR products was analyzed in a 1.5% agarose gel using RedSafe Nucleic Acid Staining Solution (iNtRON Biotechnology, Kirkland, WA) as stain and visualized by UV illumination.

**Table 1.** Primers used in multiplex PCR for detection of colistin resistance genes.

Primer name	Sequence (5′-3′)	Target gene	Size (pb)	Positive control strains	Reference
*mcr1* f^a^	AGTCCGTTTGTTCTTGTGGC	*mcr-1*	320	*mcr-*1 DTU	Rebelo *et al*. (2018)
*mcr1* r^a^	AGATCCTTGGTCTCGGCTTG				
*mcr2* f	CAAGTGTGTTGGTCGCAGTT	*mcr-2*	715	*mcr-*2 DTU	Rebelo *et al*. (2018)
*mcr2* r	TCTAGCCCGACAAGCATACC				
*mcr*3 f	AAATAAAAATTGTTCCGCTTATG	*mcr-3*	929	*mcr-*3 DTU	Rebelo *et al*. (2018)
*mcr3* r	AATGGAGATCCCCGTTTTT				
*mcr4* f	TCACTTTCATCACTGCGTTG	*mcr-4*	1116	*mcr-*4 DTU	Rebelo *et al*. (2018)
*mcr4* r	TTGGTCCATGACTACCAATG				
*mcr5* f	ATGCGGTTGTCTGCATTTATC	*mcr-5*	1644	*mcr-*5 DTU	Borowiak *et al*. (2017)
*mcr*5 r	TCATTGTGGTTGTCCTTTTCTG				

a f, forward; r, reverse.

## RESULTS

### Prevalence of colistin-resistant *E. coli* in pig farms and farm workers

Growth of typical *E. coli* colonies on MacConkey agar containing 4 mg/L colistin was observed from pigs at 53/116 (45.7%) farms and from fecal samples from workers at 21/94 (22.3%) farms. A total of 103 and 38 isolates were obtained from pig feces and human excreta, respectively and tested for resistance to colistin by the broth microdilution method. The results showed that 102 pig isolates, representing pigs at all 53 farms and 34 human isolates representing workers at 19 farms were resistant to colistin (MIC >2.0 mg/L). Thus, the confirmed farm colistin prevalence for pigs was 45.7% (53/116) and 20.2% (19/94 farms) for samples from the workers. Colistin-resistant *E. coli* isolates from workers were detected at 10 farms where isolates from pigs did not display resistance to colistin. Colistin-resistant *E. coli* were isolated from both pigs and workers at 12 farms. No geographic clustering of farms that yielded colistin-resistant *E. coli* was observed.

### Antimicrobial susceptibility of colistin-resistant *E. coli*

The MIC values for colistin-resistant *E. coli* isolates varied between 4 and 16 mg/L, confirming that isolates obtained on MacConkey plates with 4 mg/L were indeed resistant. Among the 102 pig isolates, 73/102 (71.6%) had an MIC of 4 mg/L, 25/102 (24.5%) had an MIC of 8 mg/L and the remaining 4/102 (3.9%) had an MIC of 16 mg/L. Among the 34 isolates from workers 22/34 (64.7%) showed an MIC of 4 mg/L and the remaining 12/34 (35.3%) isolates had an MIC of 8 mg/L.

Disk diffusion was used to test phenotypic susceptibility of the colistin-resistant isolates to other antimicrobials (Table 2). All *E. coli* isolates irrespective of their origin showed high resistance levels to sulfonamides (34/34; 100% of human isolates, 96/102; 94% of pig isolates), ampicillin (85%; 29/34 of human isolates, 96%; 98/102) of pig isolates), tetracycline and trimethoprim (more than 90% of isolates from both hosts), while none of the isolates were resistant to cefoxitin and amoxicillin-clavulanic acid. Low levels of resistance were observed to ceftiofur and ceftriaxone of human (1/34; 2.9%) and pig (4/102; 3.9%) isolates for both antimicrobials. All human colistin-resistant *E. coli* isolates and 98% (100/102) of pig isolates were MDR. A total of one pig isolate and two isolates from workers showed resistance to 10 antimicrobials. Due to the relative low number of isolates, 95% confidence intervals on the prevalence estimates were relatively high, and there were no significant differences between antimicrobial resistance prevalence for any of the drugs tested between isolates from pigs and farm workers. A total of 42 different phenotypic resistance profiles were observed among the colistin-resistant *E. coli* strains (Table S1, Supporting Information). Of these, nine profiles encompassing 69 pig isolates and 22 strains from workers were shared between isolates from pigs and workers, however, shared profiles between pig and human isolates on the same farm was only observed in four cases. The most common profiles were AMP; TMP; TET; STR; SUL; COL (18 isolates) and AMP; GEN; TMP; TET; STR; NAL; SUL; CIP; COL (17 isolates). A total of two colistin-resistant *E. coli*, which were found to be cephalosporin-resistant by disc diffusion testing, were positive in the ESBL synergy test.

**Table 2.** Phenotypic antimicrobial resistance of colistin-resistant *E. coli* isolates.

		Human excreta (*N* = 34 isolates)			Pig feces (*N* = 102 isolates)		
No.	Antimicrobials	No. of resistant isolates (*n*)	%	CI (0.95)	No. of resistance isolates (*n*)	%	CI (0.95)
1	AMP	29	85.3	68.9–95.0	98	96.1	90.3–98.9
2	GEN	14	41.2	24.6–59.3	52	51.0	40.9–61-0
3	TMP	31	91.2	76.3–98.1	95	93.1	86.4–97.2
4	TET	31	91.2	76.3–98.1	96	94.1	87.6–97.8
5	STR	17	50.0	32.4–67.6	69	67.6	57.7–76.6
6	NAL	16	47.1	29.8–64.9	44	43.1	33.4–53.3
7	SUL	34	100.0	89.7–100	96	94.1	87.6–97.8
8	CIP	9	26.5	12.9–44.3	31	30.4	21.7–40.3
9	CRO	1	2.9	NC	4	3.9	1.1–9.7
10	FOX	0	0.0	NC	0	0.0	NC
11	AMC	0	0.0	NC	0	0.0	NC
12	EFT	1	2.9	NC	4	3.9	1.1–9.7
13	ESBL production	0	0.0	NC	2	2.0	NC

### Prevalence of *mcr* genes

The *mcr-1* gene was detected in 97/102 (95.1%) of *E. coli* from pigs and in 31/34 (91.1%) colistin-resistant isolates from humans. The remaining eight isolates did not carry *mcr-1* genes, but did show phenotypic colistin resistance (MIC values were 4–8 mg/L) as well as resistance to several other antimicrobials including TMP, GEN, TET and SUL (Table S1, Supporting Information). One pig isolate carried both the *mcr-1* and *mcr-3* genes, whereas *mcr-2, mcr-4* and *mcr-5* genes were not detected (Table 3).

**Table 3.** Colistin resistance genes found in colistin-resistant *E. coli*.

	Isolates from pigs			Isolates from farm workers		
Colistin MIC values	Number of resistant isolates	*mcr* genes	Number with *mcr* genes (%)	Number of resistant isolates	*mcr* genes	Number with *mcr* genes (%)
4	73	*mcr-1*	69 (67.6)	22	*mcr-1*	21 (61.8)
		*mcr-3*	1^a^(1)			
		No gene	4 (3.9)		No gene	1 (2.9)
8	25	*mcr-1*	24 (23.5)	12	*mcr-1*	10 (29.4)
		No gene	1 (1)		No gene	2 (5.9)
16	4	*mcr-1*	4 (3.9)			

a One *E. coli* isolate from pig carried both *mcr-1* and *mcr-3* genes.

## DISCUSSION

The current study has documented occurrence of colistin-resistant *E. coli* in almost half of pig farms in the study area in Vietnam, and that humans working on the farms also commonly (almost one fourth of farms) harbor such bacteria. Previous studies have found highly variable (5–71.4%) carrier frequencies of colistin-resistant *E. coli* among randomly selected humans in different rural areas of Vietnam (Trung *et al*. 2017; Yamamoto 2018; Kawahara *et al*. 2019), and the carriage frequency among pig workers in the current study was well within this range. Previous studies have used growth at 2 mg/L colistin as initial selective criteria, while we have used 4 mg/L, and a direct comparison between studies is not possible. A study from Poland reported that the *mcr-1.1* variant was mostly (66.3%) found in isolates with MIC 2 mg/L (Zając *et al*. 2019), suggesting that we may have underestimated the true prevalence of colistin resistance due to the *mcr-1* gene. It would be interesting to carry out a comparison of farm workers and randomly picked human subjects using the same sampling and culture procedure in both cohorts. Results on prevalence of colistin resistance in *E. coli* seems to vary depending on concentration used for selection and the media used for isolation (Kawahara *et al*. 2019). We choose 4 mg/L as our previous studies on Danish pigs had shown that only a fraction of *E. coli* isolated on MacConkey plates with 2 mg/L were resistant in the broth microdilution assay. However, *mcr* genes turned out to be absent in that population of *E. coli* (unpublished). There is a need to agree on a standardized approach to increase the use of results for comparative purposes.

According to Vietnamese regulations, both domestic and imported veterinary products can contain colistin (MARD 2016) and colistin is assumed commonly used by Vietnamese pig farmers. Investigations in two provinces suggested that 37% of pig farmers used colistin in 2008 (Hao, Tuat and Thao 2008) and 45% of pig farmers in another province used colistin in 2015 (Toan and Luu 2015). However, reliable data on amounts and drivers of colistin use by Vietnamese pig farmers, but also poultry farmers, are lacking and should be given priority in the on-going national efforts to establish monitoring programs of antimicrobial use in livestock. In our study, reliable data on colistin use was unfortunately not available for the pig farms and correlation between actual use and resistance levels at farm level can therefore not be estimated. Colistin has been recommended for treatment of pig diseases at the dose of 100 000 IU per kg weight for 3–5 consecutive days following recommendations by the European Medicines Agency (European Medicines Agency 2016). The price of colistin sold to Vietnamese farmers is low (9–11 USD/kg) compared to the price of other antimicrobials such as amoxicillin (17–20 USD/kg). In light of the high herd prevalence of colistin resistance observed in the current study, it should be considered to regulate the use of colistin in pig farming in Vietnam in order to adhere to the WHO recommendation that HP-CIAs should not be used in livestock production (World Health Organization 2017b).

The *mcr-1* gene has spread globally in *K. pneumoniae, E. coli* and *Salmonella* spp. isolates of animal, environmental and human origin (Xavier *et al*. 2016; Dalmolin, De Lima-Morales and Barth 2018; Zhang *et al*. 2018; Ström Hallenberg *et al*. 2019; Yamamoto *et al*. 2019) mainly because it is typically located on transferable plasmids (Dalmolin, De Lima-Morales and Barth 2018; Randall *et al*. 2018). Colistin resistance in *E. coli* from pigs and workers in the current study was almost exclusively found to be associated with the presence of the *mcr-1* gene, however, we cannot rule out that strains concurrently harbor chromosomal mutations conferring resistance towards colistin. Our results corroborate other recent studies in Vietnam. These have shown that colistin resistance was due to either *mcr-1* and/or *mcr-3* in 69/70 *E. coli* isolates (MIC>2 mg/L) from rural people (Yamamoto 2018), and *mcr-1* in 60/62 colistin-resistant *E. coli* from meat and seafood (Yamaguchi *et al*. 2018), and it seems firmly established that the *mcr-1* gene is common in colistin-resistant *E. coli* isolates in Vietnam. In this situation, the widespread use of colistin in pig industry is very worrying. A total of eight isolates showing phenotypically resistance to colistin did not contain any *mcr* genes in the PCR. Lack of *mcr-1*–*5* genes in our strains showing phenotypic colistin resistance is possible. In such cases the resistance can be due to mutations into protein sequences of *pmrA/B, phoP/Q* and *mgrB* (Dagher *et al*. 2020) but also because of presence of novel *mcr* genes, e.g. *mcr-10*, not target by our PCR (Wang *et al*. 2020).

Colistin-resistant isolates were almost all shown to be MDR. The very high MDR frequency is in line with previous reports on *E. coli* from pig and humans in Vietnam. Thus, 86.7% (124/143) of *E. coli* from pigs in Vietnam have been reported to be MDR (Nhung *et al*. 2015). The resistance patterns observed did not differ significantly from resistance patterns observed in ESBL-producing *E. coli* and commensal *E. coli* in general (own unpublished results), and as such the resistance patterns described cannot be considered a particular trait of the colistin-resistant *E. coli* studied. Unnecessary prescriptions and indiscriminate use of antimicrobials have been suggested as main reasons for the high prevalence of MDR in humans in Vietnam (Le 2019).

Studies have reported a high association of the *mcr-1* gene with ESBLs genes of the CTX-M, SHV and TEM types (Dalmolin, De Lima-Morales and Barth 2018). The mcr-1 gene has also been identified in ESBL-producing and carbapenemase-producing *E. coli* from chicken or pig samples in Germany and China (Liu *et al*. 2016; Saras and Métayer 2016), and *mcr-3* has been reported in ESBL-producing *E. coli* isolates from pork (Yamaguchi *et al*. 2018) and cattle, as well (Haenni *et al*. 2018). Multiple antimicrobial resistance genes including *bla*_CTX-M_, *bla*_CMY_, *bla*_TEM_, *fosA, qnrS, floR* and *oqxAB* have been detected in ESBL-producing *E. coli* from pigs in China alongside *mcr-1* on IncHI2 plasmids. Studies have further characterized two IncHI2/ST3 plasmids with high similarity to the reference plasmid pHNSHP45–2 in *E. coli* from food-producing animals in China in which *mcr-1, oqxAB* and *bla*_CTX-M_ were co-located (Li *et al*. 2016; Liu, Song and Zou 2019). The increased findings of *mcr* genes co-located with extended-spectrum-β-lactamase and carbapenemases-producing bacteria on different plasmids demonstrate a high diversity of plasmids acting as reservoirs for *mcr* genes. Such plasmids are often transmissible and represent a great threat to public health. Fortunately, in the current study, ESBL was only detected in two colistin-resistant isolates. Colistin is typically used to treat septicemia caused by ESBL- and carbapenemase-producing Gram negative bacteria, and combinations of resistances to these drugs have high clinical impact.

In conclusion, colistin-resistant *E. coli* was found on almost half of the studied pig farms in Vietnam and also commonly in farm workers, who had close contact to pigs. Colistin resistance was found mainly to be encoded by *mcr-1* gene in both pig and human isolates. Further studies are needed to determine to what extent colistin-resistant *E. coli* from pigs are a source of colistin-resistant bacterial strains found in humans.

## Supplementary Material

xtaa003_Supplemental_File

xtaa003_Peer_review_and_author_response
