# Supplementary material for: Research note: Occurrence of mcr-encoded colistin resistance in Escherichia coli from pigs and pig farm workers in Vietnam
Source: FEMS Microbes. 2020 Oct 9;1(1):xtaa003. doi: 10.1093/femsmc/xtaa003 (PMC10117427; doi:10.1093/femsmc/xtaa003)
Supplement: xtaa003_Supplemental_File [file xtaa003_supplemental_file.docx]

**Supplementary Table. Antimicrobial susceptibility of colistin-resistant *E. coli* isolates in which *mcr* genes were not detected.**

| **No.** | **ID isolate/ ID farm** | **Sample type** | **Antimicrobial resistant profiles** |
| --- | --- | --- | --- |
| 1 | Eco16 /C12 | Pig | AMP;TMP;TET;STR;SUL;COL |
| 2 | Eco25 /E04 | Pig | AMP;GEN;TMP;TET;STR;NAL;SUL;CIP;COL |
| 3 | Eco28 /E07 | Pig | AMP;GEN;TMP;TET;NAL;SUL;COL |
| 4 | Eco34 /E13 | Pig | AMP;TET;COL |
| 5 | Eco67 /D13 | Farm-worker | AMP;TMP;TET;SUL;COL |
| 6 | Eco151 /C21 | Pig | AMP;TMP;TET;STR;SUL;COL |
| 7 | Eco177 /C17 | Farm-worker | AMP;GEN;TMP;TET;STR;NAL;SUL;COL |
| 8 | Eco82 /E07 | Farm-worker | TET;SUL;COL |

(AMP, ampicillin; TMP, trimethoprim; TET, tetracycline; STR, streptomycin; SUL, sulfonamide; COL, colistin sulfate; GEN, gentamycin; NAL, nalidixic acid; CIP, ciprofloxacin).
