# Supplementary material for: Research note: Occurrence of mcr-encoded colistin resistance in Escherichia coli from pigs and pig farm workers in Vietnam
Source: FEMS Microbes. 2020 Oct 9;1(1):xtaa003. doi: 10.1093/femsmc/xtaa003 (PMC10117427; doi:10.1093/femsmc/xtaa003)
Supplement: xtaa003_Peer_review_and_author_response [file xtaa003_peer_review_and_author_response.docx]

**Peer review comments for FEMSMC-2020-004**

Reviewer 1: Prefers to remain anonymous

The research paper by Dang *et al* reports that pig faeces on nearly half of sampled farms in a defined geographic region in North Vietnam contained colistin resistant *mcr-1* positive *E. coli*. Humans from one quarter of the sampled farms also demonstrated faecal carriage of colistin resistant *mcr-1* positive *E. coli.* Overall, the study is clear, concise and contributes to understanding the nature of agricultural AMR in Vietnam. Furthermore, it adds to global literature on the rapid emergence of colistin resistance and highlights the importance of regulating agricultural antimicrobial use, particularly in lower income countries where regulation has historically been lacking.

**General Comments**

Line 76: You mention in the introduction that data from semi-intensive farms is lacking. Could you comment on the scale of the 116 farms sampled in the study?

Line 80: Was the length of employment at the farm/amount of contact with pigs prior to sampling recorded or considered as a criterion for which human was sampled? If you have this information, you could provide a sentence describing it or disregard otherwise.

Line 120: Could you please provide a reference or clarify the DNA isolation method ‘thermo cell lysis’. Did you mean ‘thermal cell lysis’ or did you use a DNA isolation kit from Thermo-Fisher?

Sections 3.2 and 3.3 Please provide *n/total* for all quoted percentages.

Were there any instances of colisitin resistant human isolates from farms where the pig isolates did not display any resistance? If yes, please report.

I feel that value could be added to the discussion about colistin resistance and MDR by briefly referring to literature on *mcr-1* carrying plasmids. Particularly those of the HI2 family that, in contrast to X4 and I1 *mcr-1* carrying plasmids, often carry multiple other resistance determinants (including CTX-M genes) and are known to be extensively disseminated in Chinese food-production animals, particularly swine. Determining the molecular context of *mcr-1* and other resistance genes in this collection could be cited as a future direction and may be used to reveal epidemiological links on a larger geographical scale. The following three studies are most pertinent in my opinion:

1. <https://pubmed.ncbi.nlm.nih.gov/28073961>
2. <https://pubmed.ncbi.nlm.nih.gov/30179517/>
3. <https://pubmed.ncbi.nlm.nih.gov/31122100>

**Minor Comments**

Line 64/65: Please reword: The emergence of mobile colistin-resistance genes (*mcr*) on plasmids…

Line 66 and throughout: ‘*mcr-*genes’ does not require a hyphen.

Line 113: Remove double full-stop.

Line 133: Suggest reword: A total or 103 and 38 isolates were obtained from pig feces and human excreta, respectively and tested for resistance to colistin by the broth microdilution method.

Section 3.2: Grammar – ‘an MIC’ not ‘a MIC’

Line 173: English - Please change ‘very varying’ to ‘highly variable’

Line 175: Should be ‘areas’ not ‘area’

Line 185: Spelling and reword: ‘We chose 4 mg/L as our previous studies on Danish pigs…’

Line 191: Should be ‘an investigation’ not ‘and investigation’

Line 207: Should be ‘strains’ not ‘strain’

Line 217: Should be ‘patterns’ not ‘patters’

Table 2: Should be ‘No. of resistant isolates’

Please ensure consistency between Tables 2 and 3 in terms of usage of ‘strains/isolates’. I would recommend isolates.

Table 2: I assume that the CI reporting is related to CLSI phenotypic testing but there is no interpretation in results or discussion so this data could possibly be removed or provided as supplementary only.

Reviewer 2: Daria Van Tyne

The study by Dang and colleagues aims to investigate the occurrence of mcr-encoding colistin resistance in E. coli sampled from pigs and pig farm workers across Vietnam. The authors isolated 102 colistin-resistant E. coli isolates from pigs and 34 isolates from farm workers, and tested the susceptibility of each isolate against colistin and other antibiotics. The authors found that colistin-resistant E. coli were present in pigs at almost half of the sampled farms, as well as in farm workers at nearly one quarter of the farms. The data are clearly presented and the conclusions are largely supported by the data. Nonetheless the study could be improved by addressing the following comments:

1. What was the geographic distribution of the sampled farms, and was there any geographic clustering between those that yielded colistin-resistant E.coli?

2. Were farmers asked if they used colistin on their farms? If so this information should be presented. If not there appears to be some data available about colistin use in different provinces (lines 191-192). Did farms in provinces with higher colistin use show a higher prevalence of colistin-resistant E. coli?

3. How frequently were colistin-resistant E.coli-positive pig and farm worker samples isolated from the same farm? This is critical information that is currently missing from the manuscript.

4. The authors note that colistin-resistant E.coli-positive pig and farm worker isolates from the same farm rarely shared the same antimicrobial susceptibility profiles, suggesting limited transmission between pigs and farm workers. Given that the mcr-1 and mcr-3 genes are often found on plasmids, however, one could easily imagine these plasmids moving between the genetically distinct E. coli strains in the pigs vs. the workers. This should be discussed.

5. The number of colistin-resistant isolates with no resistance gene identified is small (8 isolates total in Table 3). What are the antimicrobial susceptibility profiles of these isolates? Providing some additional speculation about possible resistance genes or mutations in these isolates would be helpful.

6. Table 2: Were any of the differences between antibiotic resistance prevalence for any of the drugs tested significantly different between the pig isolates vs. farm worker isolates? If so these should be reported in Table 2; if not this should also be reported in the text.

**Author response to referee comments:**

Dear Sir/ Madam,

Thank you very much for valuable recommendations from two reviewers! I much hope that item to item response to all comments below as well as the revised version of the manuscript will make you satisfy.

I am looking for your consideration!

Reviewers' Comments to Authors:

A/ Reviewer 1:

The research paper by Dang et al reports that pig faeces on nearly half of sampled farms in a defined geographic region in North Vietnam contained colistin resistant mcr-1 positive E. coli. Humans from one quarter of the sampled farms also demonstrated faecal carriage of colistin resistant mcr-1 positive E. coli. Overall, the study is clear, concise and contributes to understanding the nature of agricultural AMR in Vietnam. Furthermore, it adds to global literature on the rapid emergence of colistin resistance and highlights the importance of regulating agricultural antimicrobial use, particularly in lower income countries where regulation has historically been lacking.

General comments
Comment: Line 76: You mention in the introduction that data from semi-intensive farms is lacking. Could you comment on the scale of the 116 farms sampled in the study?
Response: A total of 116 pig farms were sampled including 31 small-scale farms (≤ 20 pigs/farm), 66 middle-scale farms (21-99 pigs/farm), and 19 large-scale farms (≥ 100 pigs/farm). This information is now provided in Section 2.1.

Comment: Line 80: Was the length of employment at the farm/amount of contact with pigs prior to sampling recorded or considered as a criterion for which human was sampled? If you have this information, you could provide a sentence describing it or disregard otherwise.
Response: We agree that length of employment and type and frequency of contact with pigs prior to sampling are relevant factors when evaluating levels of fecal exposures and transmission between the two hosts. Unfortunately, we do not have such information about the workers that were sampled at the pig farms.

Comment: Line 120: Could you please provide a reference or clarify the DNA isolation method ‘thermocell lysis’. Did you mean ‘thermal cell lysis’ or did you use a DNA isolation kit from Thermo-Fisher?
Response: We did use a DNA isolation kit from Thermo-Fisher which has now been clarified in the revised text in Section 2.4. We apologize for this error.

Comment: Sections 3.2 and 3.3 Please provide n/total for all quoted percentages.
Response: As requested, we have now provided n/total values for the quoted percentages.

Comment: Were there any instances of colisitin resistant human isolates from farms where the pig isolates did not display any resistance? If yes, please report.
Response: Colistin-resistant E. coli isolates from workers were detected at ten farms where pigs did not yield any isolates showing colistin resistance. This information has now been added in Section 3.1.

Comment: I feel that value could be added to the discussion about colistin resistance and MDR by briefly referring to literature on mcr1 carrying plasmids. Particularly those of the HI2 family that, in contrast to X4 and I1 mcr1 carrying plasmids, often carry multiple other resistance determinants (including CTX-M genes) and are known to be extensively disseminated in Chinese food-production animals, particularly swine. Determining the molecular context of mcr1 and other resistance genes in this collection could be cited as a future direction and may be used to reveal epidemiological links on a larger geographical scale. The following three studies are most pertinent in my opinion:
1. https://pubmed.ncbi.nlm.nih.gov/28073961
2. https://pubmed.ncbi.nlm.nih.gov/30179517/
3. https://pubmed.ncbi.nlm.nih.gov/31122100

Response: We agree with the reviewer that the Discussion section can be strengthened by adding text discussing colistin resistance and MDR with reference to literature on mcr1 carrying plasmids. Thus, the following text has been incorporated into the discussion text (This response also addresses a similar comment raised by Reviewer no. 2 as shown below):
Multiple antimicrobial resistance genes including blaCTX-M, blaCMY, blaTEM, fosA, qnrS, floR and oqxAB have been detected in ESBL-producing E. coli from pigs in China alongside mcr-1 on IncHI2 plasmids (Li et al., 2017). Liu et al. (2019) further characterized two IncHI2/ST3 plasmids with high similarity to the reference plasmid pHNSHP45-2 in E. coli from food-producing animals in China in which mcr1, oqxAB, and blaCTX-M were co-located. The increased findings of mcr genes co-located with extended-spectrum-β-lactamase and carbapenemases-producing bacteria on different plasmids demonstrate a high diversity of plasmids acting as reservoirs for mcr genes. Such plasmids are often transmissible and represent a great threat to public health.

Comments (minor):
Response: We agree with all the minor comments provided below by the reviewer and have revised accordingly.

Line 64/65: Please reword: The emergence of mobile colistin-resistance genes (mcr) on plasmids…

Line 66 and throughout: ‘mcr-genes’ does not require a hyphen.

Line 113: Remove double full-stop.

Line 133: Suggest reword: A total or 103 and 38 isolates were obtained from pig feces and
human excreta, respectively and tested for resistance to colistin by the broth microdilution
method.

Section 3.2: Grammar – ‘an MIC’ not ‘a MIC’

Line 173: English - Please change ‘very varying’ to ‘highly variable’

Line 175: Should be ‘areas’ not ‘area’

Line 185: Spelling and reword: ‘We chose 4 mg/L as our previous studies on Danish pigs…’

Line 191: Should be ‘an investigation’ not ‘and investigation’

Line 207: Should be ‘strains’ not ‘strain’

Line 217: Should be ‘patterns’ not ‘patters’

Table 2: Should be ‘No. of resistant isolates’

Comment:
Please ensure consistency between Tables 2 and 3 in terms of usage of ‘strains/isolates’. I
would recommend isolates.
Response: As suggested, we have revised the two tables using the term “isolate”.

Comment: Table 2: I assume that the CI reporting is related to CLSI phenotypic testing but there is no interpretation in results or discussion so this data could possibly be removed or provided as supplementary only.
Response: We have added comments to the CI observed in the text, in the Results section.


B/ Reviewer 2:
Comments to the Author
The study by Dang and colleagues aims to investigate the occurrence of mcr-encoding colistin resistance in E. coli sampled from pigs and pig farm workers across Vietnam. The authors isolated 102 colistin-resistant E. coli isolates from pigs and 34 isolates from farm workers, and tested the susceptibility of each isolate against colistin and other antibiotics. The authors found that colistin-resistant E. coli were present in pigs at almost half of the sampled farms, as well as in farm workers at nearly one quarter of the farms. The data are clearly presented and the conclusions are largely supported by the data. Nonetheless the study could be improved by addressing the following comments:

Comment:
1. What was the geographic distribution of the sampled farms, and was there any geographic clustering between those that yielded colistin-resistant E. coli?
Response: We conducted the study in four districts in the province of Bac Ninh which is located in Northern Vietnam. Farms were randomly selected from a list of pig farms provided by the local Department of Animal Health. The districts were main areas of pig production and located closely together. The total of 116 selected pig farms were evenly distributed in the four provinces and there did not seem to be any geographic clustering between those that yielded colistin-resistant E. coli. Details on the pig farms and their selection is provided in Section 2.1 and we have added a sentence in the Results section on lack of clustering of farms that yielded colistin-resistant E. coli.

Comment:
2. Were farmers asked if they used colistin on their farms? If so this information should be presented. If not there appears to be some data available about colistin use in different provinces (lines 191-192). Did farms in provinces with higher colistin use show a higher prevalence of colistin-resistant E. coli?
Response: Our study is part of a larger project where we will collect information on use of antimicrobials, including colistin at the farms. However, questionnaire-based interviews are not really useful to collect such information as Vietnamese pig farmers often have little knowledge and memory on the types of antimicrobials they use. Farmers also rarely keep note books or otherwise keep records of antimicrobial or other veterinary drugs use at their farms. We are currently testing out alternative methods to collect information on antimicrobial use at the pig farms, e.g. placement of transparent plastic boxes where farmers and their veterinarians are asked to place any container, package, bottle etc. which had contained antimicrobials and were used at the farm. We would then at certain time intervals collect and document what were deposited in the boxes and use this as a reference point for discussion with the farmers on their antimicrobial use practices and to estimate actual antimicrobial use at the farm.

As the scarce data available on colistin use at Vietnamese pig farms have been generated mainly using questionnaire interviews and also because there are likely to be large variations in colistin use practices in different geographical areas and between different type and sizes of pig farms, we are reluctant to use any such data as a reference point for discussing our findings. The data available in Vietnam on colistin use and resistance do not allow for an analysis of whether farms with high colistin use also have higher resistance levels.

With reference to above we have revised the text in the second paragraph of the Discussion section.

Comment:
3. How frequently were colistin-resistant E. coli-positive pig and farm worker samples isolated from the same farm? This is critical information that is currently missing from the manuscript.
Response: Colistin-resistant E. coli isolates from pigs and workers at the same farm were isolated from a total of 12 farms. This information has now been added to the manuscript in Section 3.1. See also above our response to a similar comment raised by Reviewer no. 1.

Comment:
4. The authors note that colistin-resistant E. coli-positive pig and farm worker isolates from the same farm rarely shared the same antimicrobial susceptibility profiles, suggesting limited transmission between pigs and farm workers. Given that the mcr-1 and mcr-3 genes are often found on plasmids, however, one could easily imagine these plasmids moving between the genetically distinct E. coli strains in the pigs vs. the workers. This should be discussed.
Response: We agree with the reviewer and have added such text in the fifth paragraph of the Discussion section. The included new discussion text also addresses a similar request provided in a comment by Reviewer no. 1.

Comment:
5. The number of colistin-resistant isolates with no resistance gene identified is small (8 isolates total in Table 3). What are the antimicrobial susceptibility profiles of these isolates? Providing some additional speculation about possible resistance genes or mutations in these isolates would be helpful.
Response:
The antimicrobial susceptibility profiles of the eight colistin-resistant E. coli isolates that did not contain mcr genes are shown in the following table which has been provided as a Supplementary Table. All eight isolates were confirmed resistant to colistin in MIC testing (4-8 mg/L). Lack of mcr-1/2/3/4/5 genes in strains showing colistin resistance is possible. In such cases the resistance can be due to mutations into protein sequences of pmrA/B, phoP/Q, and mgrB (Dagher et al., 2020), but also novel mcr genes, e.g. mcr-10, not target by our PCR may be present in these strains (Wang et al., 2020). These explanations have now been added at the end of the third paragraph of the Discussion section.

Supplementary Table. Antimicrobial susceptibility of colistin-resistant E. coli isolates in which mcr genes were not detected.
No. ID isolate/ ID farm Sample type Antimicrobial resistant profiles
1 Eco16 /C12 Pig AMP;TMP;TET;STR;SUL;COL
2 Eco25 /E04 Pig AMP;GEN;TMP;TET;STR;NAL;SUL;CIP;COL
3 Eco28 /E07 Pig AMP;GEN;TMP;TET;NAL;SUL;COL
4 Eco34 /E13 Pig AMP;TET;COL
5 Eco67 /D13 Farm-worker AMP;TMP;TET;SUL;COL
6 Eco151 /C21 Pig AMP;TMP;TET;STR;SUL;COL
7 Eco177 /C17 Farm-worker AMP;GEN;TMP;TET;STR;NAL;SUL;COL
8 Eco82 /E07 Farm-worker TET;SUL;COL
(AMP, ampicillin; TMP, trimethoprim; TET, tetracycline; STR, streptomycin; SUL, sulfonamide; COL, colistin sulfate; GEN, gentamycin; NAL, nalidixic acid; CIP, ciprofloxacin).


Comment:
6. Table 2: Were any of the differences between antibiotic resistance prevalence for any of the drugs tested significantly different between the pig isolates vs. farm worker isolates? If so these should be reported in Table 2; if not this should also be reported in the text.
Response: There was no significantly difference in antimicrobial resistance prevalence between isolates from pigs and farm workers. This information has now been provided in the second paragraph in the Discussion section.

Best regards,
Dang Thi Thanh Son

**Peer review comments for FEMSMC-2020-004.R1**

Reviewer 1: Prefers to remain anonymous

The authors have done a great job with both sets of reviewer comments, strengthening the manuscript overall. I now believe it should be accepted with the typographical edits below.

Line 65: 'anticipated' doesn't make any sense here. Perhaps it should be 'thought to be commonly used...'

Line 148: Reword sentence. 'Thus, the farm prevalence of colistin resistance for pigs was...etc'

Line 196: Should read: '...and shown that humans working on these farms...'

Line 216: Should read: '...assumed to be commonly...'

Line 245: Should be: 'phenotypic' not 'phenotypically'

Line 249: Should be 'targeted' not 'target'

Reviewer 2: Daria Van Tyne

The revised manuscript is improved from the original submission and all my comments have been sufficiently addressed.
